# Predicting the Dielectric Properties of Nanocellulose-Modified Presspaper Based on the Multivariate Analysis Method

**DOI:** 10.3390/molecules23071507

**Published:** 2018-06-21

**Authors:** Yuanxiang Zhou, Xin Huang, Jianwen Huang, Ling Zhang, Zhongliu Zhou

**Affiliations:** 1 State Key Laboratory of Control and Simulation of Power System and Generation Equipment, Department of Electrical Engineering, Tsinghua University, Beijing 100084, China; x-huang17@mails.tsinghua.edu.cn (X.H.); zhangling15@mail.tsinghua.edu.cn (L.Z.); liangjianzhongliu@126.com (Z.Z.); 2 School of Electrical Engineering, Xinjiang University, Urumqi 830047, China; 3 National Computer Network Emergency Response Technical Team/Coordination Center of China, Beijing 100029, China; huangjw08@foxmail.com

**Keywords:** presspaper, tensile strength, breakdown, regression prediction, multivariate analysis

## Abstract

Nanocellulose-modified presspaper is a promising solution to achieve cellulose insulation with better performance, reducing the risk of electrical insulation failures of a converter transformer. Predicting the dielectric properties will help to further design and improvement of presspaper. In this paper, a multivariable method was adopted to determine the effect of softwood fiber on the macroscopic performance of presspaper. Based on the parameters selected using the optimum subset method, a multiple linear regression was built to model the relationship between the fiber properties and insulating performance of presspaper. The results show that the fiber width and crystallinity had an obvious influence on the mechanical properties of presspaper, and fiber length, fines, lignin, and nanocellulose had a significant impact on the breakdown properties. The proposed models exhibit a prediction accuracy of higher than 90% when verified with the experimental results. Finally, the effect of nanocellulose on the breakdown strength of presspaper was taken into account and new models were derived.

## 1. Introduction

Presspaper is widely used in transformers because of the advantages of excellent performance, low cost, and environmental friendliness [1,2,3]. However, the complex and severe electric field of valve winding of converter transformers causes considerable failures of existing oilpaper materials [4,5,6], threatening the reliability of the power supply. With the development of a DC ultra-high-voltage (HVDC) transmission system, such as the ±1100 kV system in China, improvement of the performance of presspaper is crucial, especially the tensile and dielectric breakdown strength. Since nanodielectrics could withstand severe conditions, nanocellulose-modified presspaper is a promising solution [7]. Predicting the performance of presspaper and nanocellulose-modified presspaper will help guide the production of high-performance presspaper, thereby reducing the risk of faults in converter transformers and improving the safety and reliability of ultra-high voltage transmission systems. 

Presently, the prediction models of mechanical and electrical properties of presspaper mainly include theoretical analysis models, simulation models, and empirical models. By physical modeling of presspaper, the simulation model obtains concrete results using finite element analysis and other numerical calculation methods. For the physical modeling of insulating paper, a two- or three-dimensional fiber network model, such as the KCL-Pakka model [8], can be built on the specific morphology of the microfibers; a simplified porous structure model of presspaper can also be established using series or series-parallel units [9]. The former is mostly used for mechanical performance simulation analysis, and the latter is mostly used for electrical performance simulation analysis. With the development of μ-CT technology, a new modeling method has emerged, namely modeling based on the actual three-dimensional structure of paper [10].

Theoretical analysis models are based on the actual physical process. There are many theoretical models for paper sheet to tensile strength, including the Cox model, the Kallmes–Bernier–Perez model, the Page model, the Allan–Neogi model, the Kane model, and the Shallhorn–Karnis model [11,12,13]. The first four models consider that the tensile strength of paper is determined by the fracture of a single fiber, while the latter two consider that it is determined by the extraction of a single fiber. The Page model [14] is the most widely used among these models. The breakdown models of presspaper mainly include the model of thermal breakdown, electric breakdown, and electromechanical breakdown. Since the breakdown process is extremely complex, these models can only give qualitative conclusions rather than quantitative values. In addition, these models do not involve the fiber characteristics and structural characteristics of presspaper.

Empirical models are mainly based on experimental results, using data analysis methods to explore the relationship between different variables. There are many empirical models on the tensile strength of paper sheet, including the linear/multiple linear regression model, partial least squares model, neural network model, support vector machine model, etc. [15,16,17]. The relationship between the breakdown strength and the thickness and the pore size of Manila paper is studied by the adaptive fuzzy logic technique [18]. Ghosh et al. used an artificial neural network model to study the relationship between partial discharge initiation, extinction voltage of solid materials, the size of defects, and the thickness of samples [19]. Generally speaking, there are few reports on an empirical model of the breakdown strength of presspaper.

Currently, there are few reports about the relationship between the properties of microfibers and the properties of presspaper. Considering that the empirical model can partly reflect the intrinsic physical relationship between variables and benefit engineering applications, it is necessary to deepen the research. In this paper, the influence of fiber physicochemical parameters on the properties of presspaper is studied by multivariate analysis. Eighteen kinds of fibers are selected to measure their physicochemical parameters, as well as the mechanical and electrical properties of the corresponding presspaper. Then the small-sample and multiple correlation problems of the test data are analyzed, and a multiple regression model of the mechanical and breakdown characteristics of presspaper is established. Finally, considering the reinforcing effect of nanocellulose, a multiple linear model of breakdown voltage of presspaper containing nanocellulose is established. This study will provide a more reliable theoretical basis for the overall optimization of presspaper properties.

## 2. Experimental

### 2.1. Materials

This paper selected 18 different raw fibers, including softwood fiber, hardwood fiber, cotton fiber, bamboo fiber, and their mixed fibers. The selected softwood fibers cover fibers from Sweden, the United States, Canada, Russia, and China. The other fibers come from China. Nanocellulose was provided by Tianjin University of Science and Technology (Tianjin, China) with a width of 10–20 nm.

### 2.2. Preparation of Presspaper Samples

The preparation of insulating paper includes four main parts: pulp soaking, refining, handsheet forming, and hot pressing. After soaking in pure water (about 3 μS/cm) for 12 h, the kraft pulp board was dispersed and refined by a Valley beater (ZT4-00, Tong, Xingping, China) to 35–40 °SR. The beating degree was determined according to ISO 5267-1. Then, the pulp suspension was transferred to a sheet former and dehydrated. Finally, hot pressing was applied to dry the wet handsheet at 115 °C under 450 N/cm^2^ for 10 min. Prepared presspaper has a thickness of ~400 μm and a grammage of about 480 g/m^2^. 

### 2.3. Morphology Characterization

Morphologies of fibers were recorded by an Olympus BX 43 optical microscope (Olympus, Tokyo, Japan). And the related parameters were tested by L&W Fiber Tester (ABB, Kista, Sweden), including fiber length, fiber width, coarseness, fines and shape coefficient. Fines which mea fines share, are the content of the fibers ≤0.2 mm.

### 2.4. Chemical Composition

Parameters of chemical composition include total lignin, holocellulose, and hemicellulose content. The total lignin is the sum of acid-insoluble lignin and acid-soluble lignin, measured according to TAPPI 222om-1998. Holocellulose is the total carbohydrate fraction (cellulose and hemicellulose) of the raw material, estimated according to Wise et al. The content of hemicellulose was evaluated by the quality of the pulp dissolved in 18 wt % NaOH solution. Ash is the quality of the residue after the paper is burned, measured according to TAPPI T211om.

### 2.5. Crystallinity, DP, and Total Charge

XRD measurements were performed by a Rigaku RINT 2000 wide angle goniometer (Rigaku, Tokyo, Japan) in the continuous scanning mode. The X-ray source is copper K-α source working at 40 kV and 200 mA. The diffraction angle (2θ) measured ranged from 10° to 50°. The resolution was 0.02°. The crystallinity index (*CrI*) was calculated by the peak height method [20]. The equation is

*CrI* = (I_002_ − I_AM_)/I_002_ × 100% (1)

where I_200_ is the height of the (002) crystalline peak and I_AM_ is the height of the amorphous halo.

Degree of polymerization (DP) is measured according to ISO 53651-2010. The total charge was determined by conductometric titration, in which the total charge of fibers is calculated based on the consumption of NaOH.

### 2.6. Tensile Strength

Tensile strength measurements were conducted by a Zwick Z005 universal testing machine (Zwick, Ulm, Germany) by the constant rate of elongation method (100 mm/min). The size of the test pieces was 150 mm × 15 mm. The initial distance between two clamps was 100 mm. For each type of presspaper, five samples were tested at room temperature with a relative humidity of ~50%.

### 2.7. Breakdown Behavior

The DC breakdown strength of samples was tested at room temperature. According to IEC 60243, a stainless steel symmetrical electrode was used in tests, and the electrode diameter was 25 mm. The size of presspaper samples was 50 mm × 50 mm. For each kind of presspaper, 10 samples were tested.

## 3. Modeling

### 3.1. Variables

Thirteen physicochemical parameters were selected to fully characterize the morphology, chemical composition, and other properties of fibers, as shown in Table 1. The shape coefficient is the ratio of fiber projection length to actual length. A larger value indicates a smaller degree of fiber bending. To facilitate the descriptive analysis in the regression equation, the physicochemical parameters are mapped to variables *x*_1_ to *x*_13_.

**Figure 1:**
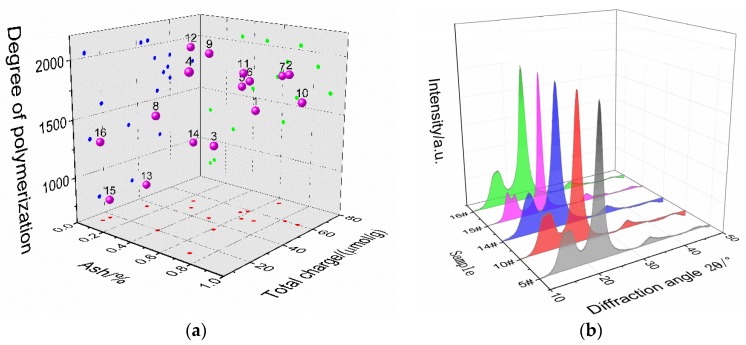
Partial results of physicochemical properties (**a**) DP, ash, and total charge; (**b**) XRD.

The 18 kinds of fibers and the presspaper samples made thereof are numbered 1 to 18 in sequence. According to Table 1, the physicochemical parameters of samples 1 to 18 were measured (shown in Table 2). In a subsequent multivariate analysis, samples 1 to 16 will be used as the training sample set and samples 17 to 18 will be used as the test sample set. There are significant changes in the value of DP, ash, and total charge. To determine whether there is a significant overlap in the characterization of these three parameters on fibers, Figure 1a gives their distribution characteristics. It can be seen that there is no obvious linear relationship, which shows that they are relatively independent. Figure 1b gives the X-ray diffraction pattern of fiber samples. The diffraction peak waveforms of the fibers are very similar overall. Only the diffraction pattern of fiber No. 15 was changed in the vicinity of 2θ = 16°, and the diffraction peak was split. Therefore, *CrI* can be used to describe the difference in crystal structure of fibers.

The mechanical properties and breakdown strength results of the 18 presspaper samples, mapped to variables *y*_1_ and *y*_2_, are shown in Table 3. There are significant differences in the mechanical and breakdown properties of the samples. The maximum values of tensile strength DC breakdown strength are 121 MPa and 23.4 kV/mm, respectively; the corresponding minimum values are 39 MPa and 9.4 kV/mm; the maximum value is 2 to 3 times the minimum value.

### 3.2. Small Sample and Multiple Correlation Problems

This paper hopes to establish a mathematical model to describe the relationship between the mechanical and electrical properties of presspaper and fiber physicochemical parameters, and realizes the prediction of presspaper performance. This requires multivariate data analysis methods. Possible methods include neural network analysis, multiple linear regression analysis, partial least-squares regression analysis, and structural equation modeling. The relationship between the input layer and the output layer of the neural network is difficult to express simply, and the model itself is quite complex; structural equations are mainly used to explore the relationship between underlying variables, while this paper studies the explicit variables of the physicochemical parameters of fibers and the mechanical and electrical properties of presspaper. There is no necessary correlation between the dependent variables (mechanical and breakdown characteristics) in the physical sense. For example, during the aging process of oilpaper insulation, the mechanical strength will decrease significantly, but the breakdown strength changes little. Consequently, this paper establishes a single-variable regression model.

Before conducting a multivariate data analysis, it is important to pay attention to the relationship between independent variables and the number of test samples. These two requirements are more stringent for multiple linear regression models, but relatively relaxed for partial least-squares regression models.

The number of sample points required to establish a multiple regression model is usually greater than the number of independent variables, recommended to be 2 to 5 times greater. Table 1 gives 13 independent variables, while the number of sample points used to build the model is only 16. Thus, the problem of few sample points needs to be solved first.

For this purpose, we can increase the number of test samples or reduce the number of variables. Considering that increasing the number of test samples is limited by many practical conditions, this paper focuses on reducing the number of variables. One method is to combine typical related analysis, filter and extract the information of each independent variable, recombine it into fewer variables, and establish the relationship between these variables and the mechanical and electrical characteristics of presspaper. This method is partial least-squares regression analysis. Another method to reduce the number of variables is to select independent variables with strong explanatory meanings to establish a multiple linear regression model. If the number of valid independent variables is 2 to 5, the problem of insufficient number of samples can be solved. 

**Figure 2:**
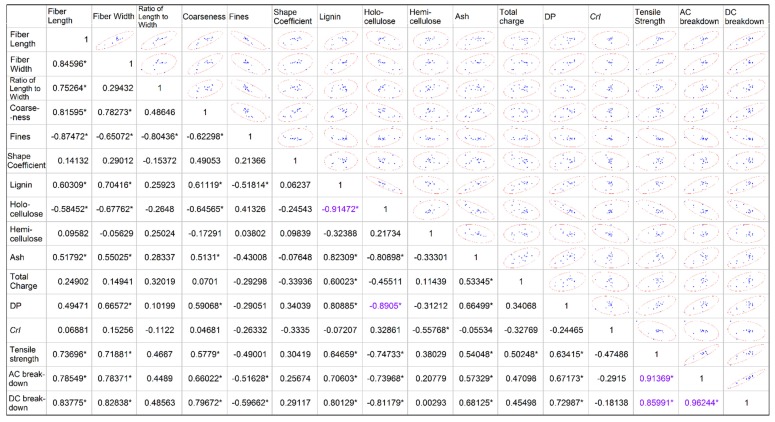
Pearson correlation coefficients among variables.

Figure 1a shows that DP, ash, and total charge are basically independent of each other, but not all of the selected physicochemical parameters. Pearson correlation coefficients were used to quantify the degree of correlation between different variables, as shown in Figure 2. It is generally believed that if the absolute value of the Pearson correlation coefficient is greater than 0.8, the correlation between the variables is very strong. All these values are maked out by “*” in Figure 2. It can be seen that there is a strong correlation between some variables in Figure 2, especially holocellulose and lignin as well as holocellulose and DP. To visually show the correlation between variables, Figure 2 also shows the scatter plots and confidence ellipses between the variables. The direction of the confidence ellipses correlates with the sign of the Pearson correlation coefficient, and the ratio of the major and minor axes correlates with the absolute value. Figure 2 indicates that the correlation between the mechanical strength and some of the fiber physicochemical parameters is not strong. Like hemicellulose, the corresponding Pearson correlation coefficient is only 0.38. For DC breakdown field strength, there is also a problem of weak correlation with some of the physicochemical parameters. It is inferred that it is not appropriate to establish the regression equation using all the physicochemical parameters as independent variables.

Partial Least Squares regression analysis is more applicable to a situation where there is a serious collinearity between variables, so that the new components extracted can effectively represent the original variables. In this problem, the Pearson correlation coefficient between many physicochemical parameters is relatively low, resulting in a recombined variable that does not include all of the information of the original independent variable. Actually, the constructed model has a poor prediction effect, and the model itself is fairly complicated, which is not conducive to the practical application. Hence, multiple linear regression is used in this paper.

For multiple linear regression, the high correlation between independent variables will lead to the difficulty and instability of regression coefficient estimation. For instance, since there is a strong correlation between hollocellulose(*x*_8_), total lignin(*x*_7_), and DP(*x*_12_), *x*_8_ will be eliminated first when conducting the multiple linear regression analysis.

To solve the small sample problem, two to five variables need to be selected from the remaining 12 physicochemical parameters. In order to select a subset that can better explain the variation of dependent variables, forward, backward, stepwise regression, and best subset selection can be used. The basis of judgment of the first three methods is to fix a test level, then calculate whether it passes the partial F test so as to conduct an independent variable screening. The choice of independent variables depends on the given test level. Therefore, this paper chooses the best subset selection method for variable selection. Based on the idea of enumeration, all possible combinations are traversed for a given number of independent variables, with the variable combinations listed with the highest goodness of fit. Then change the number of given arguments, and the best combination with different number of variables can be obtained. Considering the number of variables, *R*^2^, adjusted *R*^2^, and Mallows Cp value, the physicochemical parameters set suitable for multiple linear regression modeling could be determined. As the expected number of selected variables is 2~5, the range of given variables is 1~6 when conducting best subset selection.

Table 4 shows the best subset results of the tensile strength model for presspaper. If only one physicochemical parameter is considered, the fiber length(*x*_1_) is selected. If the number of physicochemical parameters to be considered is increased to two, the best subset is fiber width(*x*_2_) and *CrI*(*x*_13_). For a different number of variables, there is a corresponding best subset. 

Comparing the best subsets under different number of variables, we can see that with an increasing number of variables, the goodness-of-fit *R^2^* increases, indicating that the fitting effect of the regression model is improved. However, when the number of independent variables reaches a certain value, the goodness of fit that the newly introduced variable brings is very limited. If the number of variables increases from one to two, *R*^2^ increases by 32.4%; when it increases from three to four, *R*^2^ is only increased by 2.4%. Adjusted *R*^2^ can partly eliminate the increase in goodness of fit due solely to an increase in the number of variables. Only when the new variable has a certain explanatory effect on the dependent variable does adjusted *R*^2^ increase. Since fiber morphology parameters, such as fiber length and width, etc., affect the mechanical properties of the presspaper when they are introduced, the adjusted *R^2^* value increases. However, when the number of variables reaches two, increasing variables does not lead to a significant increase in the adjusted *R*^2^ value.

Mallows Cp characterize the bias and accuracy of the model. When the number of independent variables is too small, the resulting model may have a biased estimate, resulting in an excessively large value of Mallows Cp; when the number of independent variables is excessive, overfitting may occur, resulting in the value being too small. Only when the Mallows Cp value is close to the number of predictors plus the constant number can the model estimate the regression coefficients more accurately and predict new variables. In Table 3, when there is only one variable, the Mallows Cp value is 30.7, which is obviously too large; when the number of variables increases to three or more, the Mallows Cp value is too small. When the number of variables is two, the Mallows Cp value is 2.5, which is closer to 3. Considering that the goodness of fit and adjusted *R*^2^ value are relatively high, and no further significant increase occurs with increasing variables, the physicochemical parameters that are suitable for establishing the tensile strength regression model of presspaper are fiber width(*x*_2_) and crystallinity(*x*_13_). Similarly, for the AC breakdown strength regression model, the number of variables selected is three, and the selected parameters are fiber length(*x*_1_), fines(*x*_5_), and total charge(*x*_11_). The values of adjusted *R^2^* and Mallows Cp are 82.5% and 4.1, respectively. For the DC breakdown strength regression model, the number of selected variables is three, and the selected parameters are fiber length(*x*_1_), fines(*x*_5_), and total lignin(*x*_7_). The value of adjusted *R^2^* and Mallows Cp are 89.2% and 4.0, respectively.

## 4. Results and Discussion

### 4.1. Multiple Linear Regression Model for Mechanical Properties of Presspaper

**Figure 3:**
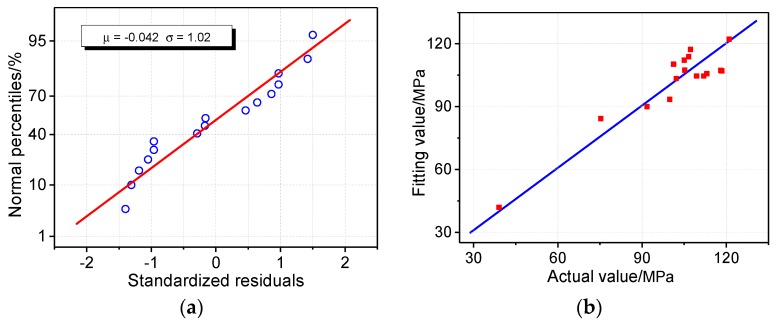
Multiple linear regression model of tensile strength of presspaper: (**a**) Normal distribution test of standardized residuals; (**b**) contrast of actual value and fitting value of regression model.

The multiple linear regression model between the tensile strength of the presspaper(*y*_1_) and fiber width(*x*_2_) and *CrI*(*x*_13_) is shown in Table 5. The goodness of fit of the model reached 87%, and predicted *R*^2^ reached 73%. The *P* value is less than 0.05 for *x*_2_ and *x*_13_, indicating their strong relationship with the tensile strength of presspaper, and it is necessary for them to be included in the model. To test the degree of collinearity among the selected variables, the variance inflation factor (VIF) is presented in Table 6. This means that there is no correlation between independent variables, if VIF is equal to 1; if VIF is greater than 10, it means that there is a strong link between independent variables; if VIF is between 1 and 5, it means there is a certain degree of correlation between independent variables, but it will not have a serious impact on the regression coefficient estimation of the model. As shown in Table 6, VIFs of *x*_2_ and *x*_13_ are very close to 1, so there is no problem of multicollinearity. The above shows that, from a statistical point of view, it is necessary and feasible that the tensile strength regression model includes both *x*_2_ and *x*_13_. To further illustrate the rationality of the model, Figure 3a shows the normal distribution of standardized residuals. Since the residuals are standardized, the μ of the normal distribution approaches 0, and σ is close to 1. The standardized residual approximates a linear trend, indicating that it follows the normal distribution, and there is no undetermined variable in the established tensile strength model. All the standardized residuals are in (−2, 2), indicating that none of the data is an abnormal observation point. Comparing the fitting values with the actual values, as designated in Figure 3b, they are very close to each other. Actually, the deviations of the fitting values from the actual values were all within ±12%, among which the deviations of the No. 7 and No. 16 samples were the largest at 9.6% and −11.9%, respectively. This indicates that the model has a good fitting effect. The above analysis indicates that the obtained model is reasonable and credible from a statistical point of view.

In terms of physical meaning, fiber width can enlarge the relative binding area between the two fibers, increasing the bond strength between different fibers [21], thus promoting the enhancement of the tensile strength of presspaper. In addition, in the sample set for this study, there is a strong correlation between fiber length and fiber width, which means that the fiber width variable conveys part of the fiber length information. According to the Page model, the fiber length is positively correlated with the tensile strength of the paper, and a large amount of experimental data also confirms this [21]. This is why fiber length is selected as the single best variable. However, the longer the fiber length implies the lower fine fiber content, which is related to beating process. Fiber width is not highly correlated with fines because refining does not substantially affect the fiber width [22]. Fines have great significance for increasing the relative binding area between fibers. Therefore, the regression coefficient of fiber width in the model is positive. An increase in crystallinity indicates a decrease in the amorphous region of fibers and a corresponding decrease in the content of lignin and hemicellulose. Since lignin and hemicellulose have a significant effect on the bond strength between fibers, the tensile strength of the presspaper decreases. In addition, because of the difference in elastic modulus between crystal and amorphous region, the risk of cavitation increases, which is the major factor of plastic deformation, affecting the tensile behavior [23]. Therefore, the regression coefficient of *CrI* in the model is negative.We used the test set to evaluate the model’s prediction results; the results are shown in Table 5. Regarding sample No. 17, the measured value is 69.7 MPa, the predicted value is 74.6 MPa, and the deviation is +9.6%; for the No. 18 sample, the actual measured value is 100.7 MPa and the predicted value is 95.8 MPa. The deviation is −4.9%. It can be seen that the predicted value is close to the actual value and the prediction interval covers the actual value. On the whole, the multiple linear regression model of the tensile strength of presspaper has an acceptable prediction performance. 

### 4.2. Multiple Linear Regression Model of DC Breakdown Strength

**Figure 4:**
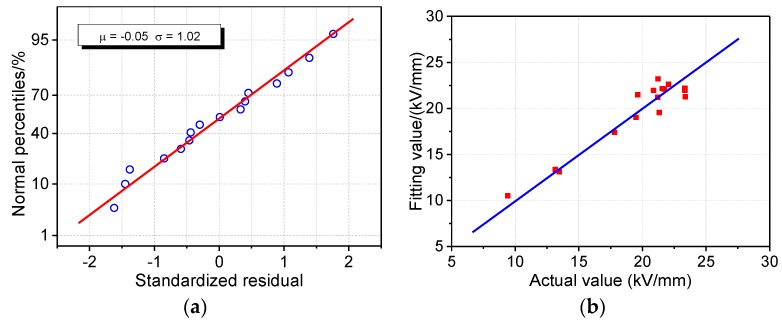
Multiple linear regression model of DC breakdown strength of presspaper: (**a**) Normal distribution test of standardized residuals; (**b**) comparison of fitting value and actual value.

The multiple linear regression model between the DC breakdown field strength of the presspaper(*y*_2_) and fiber length(*x*_1_), fines(*x*_5_), and total lignin content(*x*_7_) is shown in Table 6. The goodness of fit of the model is as high as 91%, and the predicted *R*^2^ value is 81%. All the *P* values of the independent variables are less than 0.05, and the VIF values are less than 5. This shows that the above variables have a clear explanatory effect on the DC breakdown strength, and there is no strong correlation between them. Figure 4a shows the standardized residual results of the DC breakdown strength regression model. The residual basically presents a linear trend, which illustrates that it has a normal distribution. Since standardized residuals are all located in (−2, 2), none of the data points is abnormal, indicating that this model could explain the experiment results statistically. Figure 4b shows the response characteristics of the DC breakdown strength model. The results illustrate that the fitting and actual values are very close. Specifically, the deviations of the fitting values and the actual values were all within ±12%, among which the deviations of the No. 8 and No. 15 samples were the largest at 9.0% and −11.7%, respectively. In summary, the DC breakdown strength regression model of presspaper is statistically reasonable and reliable.

In terms of physical meaning, fiber length and fines are closely related to the porous structure of the presspaper. Long fiber and relatively high fines content can moderately improve the bonding between fibers and reduce the average pore size. Breakdown of presspaper is often developed from partial discharge of internal pores [24]. According to the theory of gas discharge, smaller pores can withstand a higher electric field [24,25]. Moreover, these internal pores allow the expansion of streamer under a high electrical field, together with charge injection, extraction, and recombination, leading to bond breaking, even DC electrical tree and breakdown. [26] Therefore, longer fibers and higher fines content help to improve the breakdown strength of presspaper. In addition, fines can also improve the surface uniformity of the sheet and help the surface electric field distribute evenly [27]. Thus, the regression coefficient of *x*_1_ and *x*_5_ in the DC breakdown model is positive. 

Due to the presence of negative groups such as carboxyl groups on the surface of fibers, fibers are electronegative [28]. Lignin contains a large number of carboxyl groups and is one of the major sources of fiber charges. Therefore, its impact on the breakdown field strength is similar to that of total charge. Figure 2 also suggests that there is a strong correlation between lignin content and total charge. There is no related report on the effect of total charge on the breakdown properties of presspaper. This study found that an appropriate increase in the total charge has a positive effect on the breakdown strength. A possible explanation is that the negatively electric groups on the cellulose surface, such as carboxyl, carbonyl, etc., can be seen as a chemical defect that provides small dipole moments that can act as shallow charge traps (typically ~0.3 eV) [7]. Moderate traps can trap carriers and restrict their transport, suppressing internal discharges to a certain extent, thereby promoting the improvement of the breakdown strength [29]. This is similar to the fact that adding proper nano-additives can increase the breakdown strength because nanoparticles introduce new traps that can limit the movement of carriers [30,31,32]. Thus, the sign of the regression coefficient of x_12_ is positive in the model. On the other hand, lignin helps to increase the bonding strength between fibers and restrict the internal discharge. Therefore, the regression coefficient of the total lignin content is positive.

As shown in Table 6, for the No. 17 sample, the measured breakdown strength is 15.5 kV/mm, and the predicted field strength is 15 kV/mm with a deviation of −3.2%. For sample No. 18, the measured breakdown strength is 18 kV/mm, and the predicted strength is 17.7 kV/mm with a deviation of −1.7%. This shows that the regression model of DC breakdown strength of presspaper has an excellent prediction performance.

### 4.3. Multiple Linear Regression Model of Breakdown Strength Considering the Nanocellulose Reinforcing Effect

Existing studies have shown that the addition of nanocellulose in presspaper can effectively improve the performance of presspaper, especially the addition of cationic nanocellulose (CNFC) [33]. One explanation is that the interface developed between nanoparticles and the cellulose matrix provides deep traps for charge carriers, thereby enhancing insulating performance [7]. So far, we have used a multiple linear regression model to explore the relationship between presspaper properties and fiber physicochemical parameters. Furthermore, we hope to introduce nanocellulose modification in the model. This will allow the model to include both micron-scale fibers and nanoscale fibers, which will help enrich the connotation of the model and increase the value of application. By analogy with fines, this paper regards the content of nanocellulose, denoted as *x*_14_, as a physicochemical parameter of micro-fibers. Firstly, a linear regression model is established between the performance of presspaper and the content of nanocellulose. The constant term of the model is the performance of the presspaper without nanocellulose, then the previously obtained multiple linear regression model can be substituted, making the new model contain the reinforcing effect of nanocellulose.

**Figure 5:**
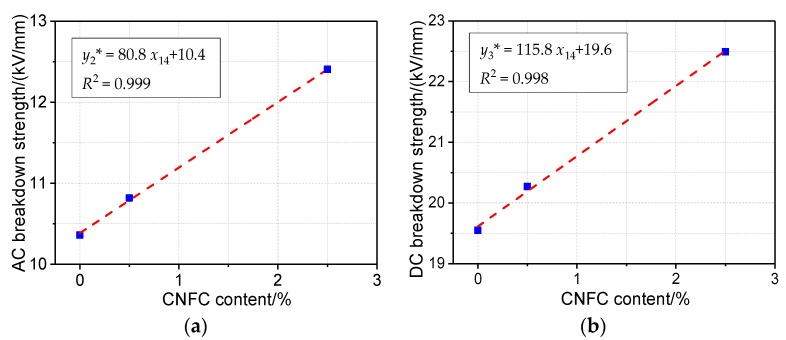
(**a**) Regression model of AC breakdown strength and CNFC content of presspaper; (**b**) regression model of DC breakdown strength and CNFC content of presspaper.

With an increasing amount of nanocellulose added to presspaper, the performance of presspaper exhibits a clear, non-linear relationship [34]. When the CNFC content does not exceed 2.5 wt %, there is an obvious linear relationship between the breakdown strength and the nanocellulose content of presspaper. Figure 5a,b give a linear regression model between the AC and DC breakdown strength and the nanocellulose content, respectively. For DC breakdown strength, the model is:

*y*_2_* = 115.8*x*_14_ + 19.6 (2)

The *R*^2^ value reached 0.998. The regression coefficient of nanocellulose *x*_14_ was 115.8, slightly higher than its coefficient in the AC breakdown regression model (*y*_3_*), which is:

*y*_3_* = 80.8*x*_14_ + 10.4 (3)

The goodness of fit *R*^2^ reached 0.999. The constant of the regression model is 10.4, which means that when the presspaper does not contain nanocellulose, the AC breakdown strength is 10.4 kV/mm. 

A multiple linear regression model for DC breakdown strength of presspaper containing reinforcing effect of nanocellulose is obtained by combining multiple regression equations in Equation (3) and Table 6:

*y*_2_* = 10.1*x*_1_ + 0.52*x*_5_ + 0.54*x*_7_ + 115.8*x*_14_ − 7.7 (4)

A regression model has been built to describe the correlation between fiber physicochemical parameters and AC breakdown strength of presspaper without nanocellulose. Combining the multiple regression equations in Equation (2) and this regression model, a multiple linear regression model for AC breakdown strength of presspaper containing the reinforcing effect of nanofibers was obtained as follows:

*y*_3_* = 7.13*x*_1_ + 0.41*x*_5_ + 0.041*x*_12_ + 80.8*x*_14_ − 7.6 (5)

It should be noted that *x*_14_ in Equations (4) and (5) should not exceed 2.5%. 

## 5. Conclusions

With the development of HVDC, the need for an environmentally-friendly and high-performance insulation paper that can withstand a higher electric field and mechanical stress has attracted research interest. This paper explores the effect of microfiber characteristics on tensile strength and DC breakdown field strength of presspaper:

1. A multiple linear regression model between tensile strength and fiber width variable and crystallinity variable was obtained. The goodness of fit was 87%, and the prediction accuracy of the test samples reached more than 90%. Multiple linear regression models were established for DC breakdown strength of presspaper. The prediction accuracy of the model for testing samples is more than 95%.
2. Multiple linear regression models of AC and DC breakdown strength of presspaper considering the reinforcing effect of nanocellulose were established. The model for AC breakdown strength is *y*_2_* = 7.13*x*_1_ + 0.41*x*_5_ + 0.041*x*_11_ + 80.8*x*_14_ − 7.6. The model for DC breakdown strength is *y*_2_* = 10.1*x*_1_ + 0.52*x*_5_ + 0.54*x*_7_ + 115.8*x*_14_ − 7.7. Among them, *x*_1_, *x*_5_, *x*_7_, *x*_11_, and *x*_1__4_ represent fiber length, fines, total lignin content, total charge, and nanocellulose content, respectively.

It should be noted that characteristics of the three-dimensional structure of paper, such as fiber orientation and paper thickness, also affect the performance of presspaper, hence further investigation may consider these factors. Moreover, commonly used nano-additives also include metal oxides, graphene, etc. and the influence of this factor needs to be elucidated so that this approach can predict other nanocomposite systems.

## Figures and Tables

**Table 1:** Variables of physicochemical parameters.

Physicochemical Parameters	Unit	Variable	Physicochemical Parameters	Unit	Variable
Fiber length	mm	*x*_1_	Holocellulose	%	*x*_8_
Fiber width	μm	*x*_2_	Hemicellulose	%	*x*_9_
Ratio of length to width	−	*x*_3_	Ash	%	*x*_10_
Coarseness	μg/m	*x*_4_	Total charge	μmol/g	*x*_11_
Fines	%	*x*_5_	DP	−	*x*_12_
Shape coefficient	%	*x*_6_	*CrI*	%	*x*_13_
Lignin	%	*x*_7_			

**Table 2:** Test results of physicochemical properties of fibers.

Sample	*x*_1_	*x*_2_	*x*_3_	*x*_4_	*x*_5_	*x*_6_	*x*_7_	*x*_8_	*x*_9_	*x*_10_	*x*_11_	*x*_12_	*x*_13_
1	2.06	31.8	65	207	5	84.3	10.2	89.8	8.6	0.70	52	1573	90.6
2	2.33	31.2	75	160	3.3	83.3	9.4	90.6	8.2	0.83	60	1877	91.2
3	2.52	31.3	81	215	2.5	84.8	7.6	92.4	10.5	0.61	34.7	1328	91.6
4	2.38	34.7	69	208	3.2	84.6	6.4	90.3	8.8	0.71	12	2038	91.5
5	2.36	31.6	75	196	2.1	84.2	8.8	91.3	9.4	0.56	56	1730	91.2
6	2.02	31.1	65	182	5	84.2	7.8	92.2	9.2	0.59	58.7	1772	90.9
7	2.29	31	74	185	4.1	82.7	8.4	91.7	10.5	0.65	74.7	1775	90.9
8	2.43	30.9	79	219	2.8	85	5.4	91.7	8.8	0.40	20	1583	91.2
9	2.24	33.5	67	178	3.3	85.3	10.2	89.7	10.0	0.49	42.7	2038	90.1
10	1.71	28.2	61	113	7.7	80.2	10.0	90.1	8.1	0.91	60	1664	91.3
11	2.29	31.3	73	184	4.5	82.9	8.1	91.6	9.5	0.63	50.7	1877	90.6
12	2.18	32.4	67	170	4.6	83.6	9.7	90.3	9.0	0.32	46.7	2046	90.7
13	2.20	30.9	71	118	4	82.2	1.5	96.4	14.2	0.11	36	804	91.2
14	1.51	19.4	78	102	8.4	82.4	2.1	94.1	12.0	0.26	53.3	1156	86.8
15	1.45	23.2	62	108	5.7	80.6	1.1	98.6	6.5	0.08	17.3	763	96
16	0.98	23.2	42	105	20.7	86.7	0.6	95.8	9.9	0.08	13.3	1298	89.8
17	1.74	27.2	64	145	5.35	82.4	4.4	95.4	7.9	0.33	38	1268	93.5
18	1.67	27.4	59	150	11.4	85.5	4.7	93.5	9.6	0.32	34.7	1514	90.5

**Table 3:** Mechanical properties and breakdown strength of presspaper samples.

Sample	Tensile Strength *y*_1_ (MPa)	DC Breakdown Strength *y*_2_ (kV/mm)	Sample	Tensile Strength *y*_1_ (MPa)	DC Breakdown Strength *y*_2_ (kV/mm)
1	105	21.2	10	100	19.5
2	113	22.1	11	101	23.3
3	102	21.2	12	107	20.9
4	107	19.6	13	112	17.8
5	118	23.3	14	92	13.5
6	105	21.3	15	39	9.4
7	118	21.7	16	75	13.2
8	109	23.4	17	70	15.5
9	121	21.6	18	101	18.0

**Table 4:** Best subset results for the tensile strength model of presspaper.

Number of Variables	*R* ^2^	Adjusted *R*^2^	Mallows Cp	Selected Variables
1	54.3	51	30.7	*x*_1_
2	86.7	84.6	2.5	*x*_2_, *x*_14_
3	90.7	88.4	0.7	*x*_2_, *x*_11_, *x*_13_
4	93.1	90.6	0.4	*x*_1_, *x*_3_, *x*_11_, *x*_13_
5	94.4	91.7	1.2	*x*_1_, *x*_3_, *x*_4_, *x*_11_, *x*_13_
6	95.6	92.7	2.1	*x*_1_, *x*_2_, *x*_3_, *x*_5_, *x*_6_, *x*_13_

**Table 5:** Multiple linear regression model of tensile strength of presspaper.

Model Information			Performance of the Prediction			
Independent variable	*P*	VIF	No.	Actual value	Predicted value	Predicted interval
*x*_2_	0.000	1.02	17	69.7	74.6	(55.7, 93.6)
*x*_13_	0.000	1.02	18	100.7	95.8	(78.1, 113.5)
*y*_1_ = 608 + 3.89*x*_2_ − 6.83*x*_13_						
*R*^2^ = 87%, predicted *R*^2^ = 73%						

**Table 6:** Multiple linear regression model of DC breakdown strength of presspaper.

Model Information			Performance of the Prediction			
Independent variable	*P*	VIF	No.	Actual value	Predicted value	Predicted interval
*x*_1_	0.000	4.90	17	15.5	15	(11.8, 18.3)
*x*_5_	0.008	4.26	18	18	17.7	(14.4, 20.9)
*x*_7_	0.001	1.57				
*y*_2_ = −7.7 + 10.1*x*_1_ + 0.52*x*_5_ + 0.54*x*_7_						
*R*^2^ = 91%, predicted *R*^2^ = 81%						

## References

[1] El-Saied H., El-Meligy M.G., Mohamed S.H., Abd El-Mongy S. (2012). Electrical insulated paper from cotton linter. Carbohydr. Polym..

[2] Fahmy T.Y.A., El-Meligy M.G., Mobarak F. (2008). Introducing deinked old newsprint as a new resource of electrical purposes paper. Carbohydr. Polym..

[3] Prevost T.A., Oommen T.V. (2002). Cellulose insulation in oil-filled power transformers: Part I—History and development. IEEE Electr. Insul. Mag..

[4] CIGRE Joint Working Group A2/B4. 28 (2010). HVDC Converter Transformers Design Review, Test Procedures, Ageing Evaluation and Reliability in Service.

[5] CIGRE Advisory Group B4.04 (2015). HVDC LCC Converter Transformers-Converter Transformers Failure Survey Results from 2003 to 2012.

[6] Zhou Y., Huang M., Sun Q., Sha Y., Jin F., Zhang L. (2013). Space charge characteristics in two-layer oil-paper insulation. J. Electrost..

[7] Pourrahimi A.M., Olsson R.T., Hedenqvist M.S. (2018). The Role of Interfaces in Polyethylene/Metal-Oxide Nanocomposites for Ultrahigh-Voltage Insulating Materials. Adv. Mater..

[8] Nilsen N., Zabihian M., Niskanen K. (1998). KCL-PAKKA: A tool for simulating paper properties. Tappi J..

[9] Morgan V.T. (1998). Effects of frequency, temperature, compression, and air pressure on the dielectric properties of a multilayer stack of dry kraft paper. IEEE Trans. Dielectr. Electr. Insul..

[10] Siltanen H. (2012). X-Ray Computed Tomography and Numerical Analysis of Water-Saturated Porous Materials. Master’s Thesis.

[11] Hubbe M.A. (2014). Prospects for maintaining strength of paper and paperboard products while using less forest resources: A review. Bioresources.

[12] He J. (2005). Quantitative Study of Paper Structure at the Fibre Level for Development of a Model for the Tensile Strength of Paper. Ph.D. Thesis.

[13] Carlsson L.A., Lindstrom T. (2005). A shear-lag approach to the tensile strength of paper. Compos. Sci. Technol..

[14] Page D.H. (1969). A theory for tensile strength of paper. Tappi.

[15] Banavath H.N., Bhardwaj N.K., Ray A.K. (2011). A comparative study of the effect of refining on charge of various pulps. Bioresour. Technol..

[16] Ciesielski K., Olejnik K. (2014). Application of neural networks for estimation of paper properties based on refined pulp properties. Fibres Text. East. Eur..

[17] Johansson A. (2016). Correlations between Fibre Properties and Paper Properties. Master’s Thesis.

[18] Mohanty S., Ghosh S. Breakdown voltage modeling of Manila paper in the presence of cavities under AC and DC conditions by adaptive fuzzy logic technique. Proceedings of the 2009 International Conference on Power Systems.

[19] Ghosh S., Kishore N.K. (2002). Modelling of partial discharge inception and extinction voltages of sheet samples of solid insulating materials using an artificial neural network. IEE Proc. Sci. Meas. Technol..

[20] Park S., Baker J.O., Himmel M.E., Parilla P.A., Johnson D.K. (2010). Cellulose crystallinity index: Measurement techniques and their impact on interpreting cellulase performance. Biotechnol. Biofuels.

[21] Wimmer R., Downes G.M., Evans R., Rasmussen G., French J. (2002). Direct effects of wood characteristics on pulp and handsheet properties of Eucalyptus globulus. Holzforschung.

[22] Huang J., Zhou Y., Dong L., Zhou Z., Liu R. Effect of pulp refining on mechanical and electrical properties of insulating presspaper. Proceedings of the 2016 IEEE Conference on Electrical Insulation and Dielectric Phenomena (CEIDP).

[23] Liu D., Pallon L.K.H., Pourrahimi A.M., Zhang P., Diaz A., Holler M., Schneider K., Olsson R.T., Hedenqvist M.S., Yu S. (2017). Cavitation in strained polyethylene/aluminium oxide nanocomposites. Eur. Polym. J..

[24] Dissado L.A., Fothergill J.C. (1992). Electrical Degradation and Breakdown in Polymers.

[25] Husain E., Nema R.S. (1982). Analysis of Paschen curves for air, N_2_ and SF_6_ using the Townsend breakdown equation. IEEE Trans. Electr. Insul..

[26] Pallon L.K., Nilsson F., Yu S., Liu D., Diaz A., Holler M., Chen X.R., Gubanski S., Hedenqvist M.S., Olsson R.T. (2017). Three-Dimensional Nanometer Features of Direct Current Electrical Trees in Low-Density Polyethylene. Nano Lett..

[27] Murata M., Nakata K. (1977). Influence of the fines on the dielectric and tensile breakdown of oil-impregnated paper. IEEE Trans. Electr. Insul..

[28] Wagberg L. (2000). Polyelectrolyte adsorption onto cellulose fibres—A review. Nordic Pulp Paper Res. J..

[29] Murakami Y., Nemoto M., Okuzumi S., Masuda S. (2008). DC conduction and electrical breakdown of MgO/LDPE nanocomposite. IEEE Trans. Dielectr. Electr. Insul..

[30] Tanaka T., Kozako M., Fuse N., Ohki Y. (2005). Proposal of a multi-core model for polymer nanocomposite dielectrics. IEEE Trans. Dielectr. Electr. Insul..

[31] Nelson J.K. (2010). Dielectric Polymer Nanocomposites.

[32] Liao R., Zhang F., Yuan Y., Yang L., Liu T., Tang C. (2012). Preparation and electrical properties of insulation paper composed of SiO_2_ hollow spheres. Energies.

[33] Huang J., Zhou Y., Dong L., Zhou Z., Liu R. (2017). Enhancement of mechanical and electrical performances of insulating presspaper by introduction of nanocellulose. Compos. Sci. Technol..

[34] Huang J., Zhou Y., Dong L., Zhou Z., Zeng X. (2017). Enhancing insulating performances of presspaper by introduction of nanofibrillated cellulose. Energies.

